# Sustained virological response is associated with clearance of hepatitis C virus RNA and a decrease in hepatitis C virus antibody

**DOI:** 10.1111/j.1478-3231.2008.01918.x

**Published:** 2009-04

**Authors:** Sarah Maylin, Michelle Martinot-Peignoux, Marie-Pierre Ripault, Rami Moucari, Ana Carolina Cardoso, Nathalie Boyer, Nathalie Giuily, Corinne Castelnau, Michelle Pouteau, Tarik Asselah, Marie Hélène Nicolas-Chanoine, Patrick Marcellin

**Affiliations:** 1 Service de Microbiologie, Hôpital BeaujonClichy, France; 2 INSERM, U-773, Centre de Recherche Biomédicale Bichat-Beaujon CRB3, Université Paris VII, Hôpital BeaujonClichy, France; 3 Service d'Hépatologie, Hôpital BeaujonClichy, France

**Keywords:** HCV antibody, pegylated interferon, residual infection, RIBA-HCV, transcription-mediated amplification (TMA)

## Abstract

**Background/Aim:**

Viral eradication in chronic hepatitis C patients with sustained virological response (SVR) after interferon (IFN) therapy remains controversial.

**Methods:**

During a long-term follow-up study, 157 patients with SVR to IFN-α-2b-based therapy were investigated with a transcription-mediated amplification (TMA) assay in serum. The hepatitis C virus (HCV) antibody was assessed by measuring the optical density (OD) (Axsym HCV v3.0) and the semiquantitative titres (RIBA HCV v3.0) of the HCV antibodies directed against the core, NS3, NS4 and NS5 proteins. A control group included 23 untreated patients with persistently normal serum alanine aminotransferase and detectable serum HCV-RNA.

**Results:**

The median duration of follow-up was 4.0 (0–10) years. Serum HCV-RNA remained undetectable in all patients. The mean HCV antibody OD were 93 ± 19 and 45 ± 21 before therapy and in the last available serum sample respectively (*P*=0.001). There was a marked decrease in the HCV antibodies directed against the NS3, NS4 and NS5 proteins (*P*=0.001), while the core protein titre remained strongly positive. The 23 control patients were followed for a median of 5 (2–14) years. The mean HCV antibody OD were 65 ± 14 and 64 ± 19 in the first and the last measurements, respectively (NS), and HCV antibody titres for structural and non-structural proteins remained unchanged.

**Conclusion:**

This long-term study evaluating 157 patients demonstrated that SVR assessed by TMA is durable, and HCV antibodies were markedly decreased (mainly those directed against the non-structural proteins), emphasizing an absence of ongoing infection. These results strongly suggest that HCV infection cured in patients who achieve an SVR.

The goal of antiviral therapy in patients with chronic hepatitis C virus (HCV) infection is to attain a sustained virological response (SVR), resulting in viral eradication in most patients (1). In the past decade, antiviral therapy for chronic hepatitis C has markedly improved with the addition of ribavirin and pegylated interferon (PEG-IFN) (2, 3), resulting in a significant improvement in SVR rates. When patients achieve SVR there is a very low risk of virological relapse (4, 5). Treatment-induced viral eradication is associated with an improvement in liver histological lesions. Several studies have reported an improvement in liver fibrosis in patients successfully treated with IFN-based therapy (6–8).

Although the liver is the main site of viral replication, HCV may also be found in extrahepatic locations such as peripheral blood mononuclear cells (PBMC) (9). Persistent viral RNA in the PBMC of patients who have either spontaneously cleared serum HCV-RNA or have cleared it after anti-HCV therapy has been reported (10–12). Conversely, two recent studies (8–13) have reported the absence of detectable HCV-RNA in the PBMC and/or the liver in patients with SVR achievement after antiviral therapy.

However, although viral eradication is well documented in patients with SVR, changes in antibodies to HCV status remain unclear. The diagnosis of resolved hepatitis C infection is based on the detection of the HCV-specific antibody and the absence of detectable serum HCV-RNA. Complete or partial seroconversion of the HCV antibody is characterized by a progressive non-synchronized loss of these antibodies (14, 15). Several studies have been performed to define the features of the humoral immune response to HCV infection; only a few small studies have focused on the dynamics of change in HCV-specific antibodies in relation to treatment-induced viral eradication (16–18).

The aim of the present study was to investigate the dynamics of change in various HCV antibodies in patients with chronic hepatitis C who achieved SVR after antiviral therapy.

## Methods

### Patients

A total of 1278 patients with histologically proven chronic hepatitis C received IFN-α-2b-based therapy, from 1990 to 2006, in our outpatient clinic. Chronic hepatitis C was defined as the presence of the HCV antibody, detectable serum HCV-RNA and elevated (twice the upper limit of normal) serum alanine aminotransferase (ALT) levels. SVR was defined as normal ALT and undetectable serum HCV-RNA with transcription-mediated amplification (TMA), at the end of treatment and after 6 months post-treatment follow-up. In a previous study (8), 344 patients with SVR were evaluated for residual HCV-RNA in the liver and PBMC and liver histology improvement. In the present study, a subgroup of 157 patients were evaluated for a long-term follow-up of post-treatment HCV antibodies.

Inclusion criteria were SVR prospectively assessed in our centre with TMA (available since January 2002) or an available frozen serum sample for retrospective measurement with TMA (before 2002). This study was approved by the University Ethics Committee and performed in accordance with the principles of the Declaration of Helsinki.

### Control group

A control group of 23 untreated patients with persistently normal serum ALT and ongoing infection (detectable serum HCV-RNA) were studied for HCV antibody follow-up.

### Follow-up

Serum HCV-RNA and serum ALT levels were measured every 6 months during the first 3 years of follow-up and every other year thereafter.

### Hepatitis C virus RNA detection

Hepatitis C virus RNA was detected qualitatively with TMA (VERSANT™ HCV RNA Qualitative assay; Siemens Medical Solution Diagnostics; sensitivity <9.6 IU/ml, Eragny, France).

### Hepatitis C virus antibody detection

Hepatitis C virus antibody measurement was performed with the enzyme immunoassay HCV version 3.0 AXSYM (Abbott Diagnostics Division, Wiesbaden, Germany) allowing detection of the antibody against HCV core NS3 fusion protein and non-structural proteins (c200, c100, NS5). Results were expressed as optical density (OD) according to the manufacturer's instructions. For each patient, the result is expressed in ratio (%); ‘OD generated by the last available serum sample/OD generated at baseline’. The ratio of decrease of HCV antibodies was defined as the difference between the baseline OD and the last measurement OD divided by the baseline OD. Because the duration between the two measurements was different, we calculated an annual decrease by dividing the ratio by the interval of time between the two measurements.

The amount and profile of the HCV antibody was confirmed with the semiquantitative CHIRON-RIBA HCV 3.0 SIA assay (Ortho clinical Diagnostics, Illkirch, France). This immunoblot assay measures antibodies directed to both structural proteins (core Ag, c22 synthetic peptide) and non-structural proteins (NS3 Ag, c33c recombinant protein; NS4 Ag, mixed c5-1-1 and c100 peptides; and NS5 Ag recombinant protein). The intensity of the coloured bands is proportional to the amount of bound antibody and is graded as 0 (none), 1+ to 4+, according to the manufacturer's instructions.

### Statistical analysis

Quantitative variables were expressed as means ± standard deviation (SD) or median with range. Statistical methods for qualitative data included the *χ*^2^ test and the anova test for quantitative data. A two-sided *P* value of <0.05 was considered statistically significant.

## Results

### Patients

The baseline demographical, clinical, virological and histological characteristics of the 157 patients are shown in Table 1. The mean duration of follow-up was 4.6 ± 2.1 years (median 4.0; range 0–10 years). The patients contributing data are shown in Table 2.

**Table 2:** Patients contributing data at each time point during follow-up

		0*	1†	2†	3†	4†	5†	6†	7–9†	≥10†
Follow-up	EOT	*n*‡	*n*‡	*n*‡	*n*‡	*n*‡	*n*‡	*n*‡	*n*‡	*n*‡
Serum HCV-RNA (*n*=278)	–	278	278	243	202	154	91	59	39	4
HCV antibody (*n*=157)§¶	66	14	7	12	27	35	23	9	28	2

* Inclusion in the study (6 months after treatment cessation).

† Years after inclusion.

‡ Number of patients tested at this time point.

§ Number of patients available for this data.

¶ Sixty-six patients had an additional testing at the end of treatment.

EOT, end of treatment; HCV, hepatitis C virus.

**Table 1:** Baseline patients characteristics

	Patients with SVR	Control
Male, *n* (%)	118 (75)	5 (17)
Age (years)		
Mean ± SD	46 ± 11	45 ± 10
Range	20–78	27–64
ALT (IU/ml)		
Mean ± SD	113 ± 75	26 ± 8
Range	45–350	11–40
Anti-HCV OD		
Mean ± SD	92 ± 19	65 ± 14
Range	28–125	43–106
Mode of infection (*n*, %)		
Blood transfusion	36 (23)	6 (26)
Injection drug use	52 (33)	6 (26)
Unknown	69 (45)	11 (48)
HCV-RNA		
Median (log_10_ IU/ml)	5.537	5.215
Range	2.259–7.532	3.602–6.000
HCV genotype (*n*, %)		
1	68 (43)	13 (56)
2	25 (16)	3 (13)
3	47 (30)	5 (22)
4–5	17 (11)	2 (9)
Fibrosis stage (*n*, %)*		
F0–F1	54 (37)	
F2	55 (37)	Not available
F3	24 (17)	
F4	13 (9)	
Pretreatment status (*n*, %)		
Naïve	94 (60)	–
Non-responders	26 (17)	–
Relapsers	37 (23)	–
Treatment regimens (*n*, %)		
IFN-α-2b†	12 (8)	–
IFN-α-2b plus RBV‡	24 (15)	–
PEG-IFN-α-2b plus RBV§	121 (77)	–

* Liver histology was graded according to the METAVIR scoring system: F0, no fibrosis; F1, portal fibrosis without septa; F2, portal fibrosis with rare septa; F3, numerous septa without cirrhosis; and F4, cirrhosis.

† Intron A, at a dose of 3 million units three times a week for 6 months.

‡ Intron A in combination with ribavirin (Rebetol; Schering Plough Research Institute, Kenilworth, NJ, USA), at a dose of 3 million units three times a week in combination with ribavirin at a dose of 800–1200 mg/day according to body weight.

§ PEG-Intron (Schering Plough Research Institute) at a dosage of 1.5 μg/kg body weight per week, and ribavirin (Rebetol; Schering Plough Research Institute) at a dosage of 800–1200 mg/day.

ALT, alanine aminotransferase; HCV, hepatitis C virus; IFN, interferon; OD, optical density; RBV, ribavirin; SD, standard deviation; SVR, sustained virological response.

### Clinical outcomes

Serum ALT levels remained normal in 147/157 (94%) patients. In 10 patients (6%), serum ALT levels fluctuated between normal and twice the upper limit of normal (three patients were overweight, one consumed excessive amounts of alcohol, two had diabetes, one had active chronic hepatitis B, one had steatosis and two had no cofactors). None of the 14 patients with cirrhosis at baseline developed decompensated cirrhosis. All 157 patients were alive at the end of follow-up.

### Virological outcomes

Serum HCV-RNA remained undetectable in all the serum samples tested during follow-up, indicating that none of the patients relapsed.

### Hepatitis C virus antibody measurement

**Fig. 1:**
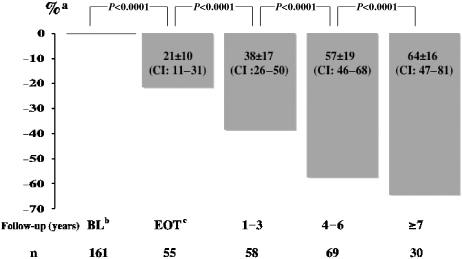
Dynamics changes in hepatitis C virus (HCV) antibody in 157 patients during a long-term follow-up after sustained virological response (SVR) to interferon-based therapy. ^a^Expressed as ratio (%) optical density (OD) generated by the last available serum sample/OD generated at baseline. ^b^Baseline. ^c^End of treatment.

Hepatitis C virus antibody measurements were performed in all the patients when treatment began, and on the last available serum sample during follow-up, 66 patients had an end-of-treatment measurement. The mean OD was 92 ± 19 and 45 ± 2 in the pretreatment and end-of-follow-up serum samples respectively (*P*=0.0001). The dynamics of OD change according to the delay between baseline and the last measurement is shown in Figure 1.

**Fig. 2:**
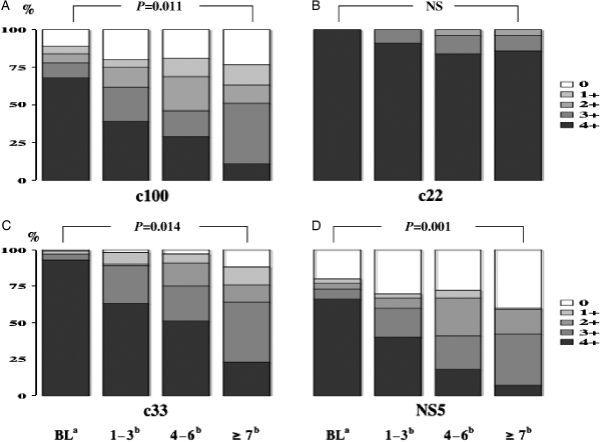
Changes in semiquantitative hepatitis C virus (HCV) antibody titres [recombinant immunoblot assay (RIBA)] measured before therapy and at the end of a long-term follow-up in 157 patients successfully treated with interferon-based therapy. ^a^Baseline. ^b^Years. (A) HCV antibody directed against NS4 protein (c100). (B) HCV antibody directed against core protein (c22). (C) HCV antibody directed against NS3 protein (c33). (D) HCV antibody directed against NS5 protein (NS5). During the follow-up, 3, 10 and 26% of NS3, NS4 and NS5 bands became undetectable respectively.

There was a significant decrease in recombinant immunoblot assay (RIBA)-HCV titres and profiles of antibodies directed against the NS3, NS4 and NS5 proteins during the follow-up (*P*=0.01), while antibodies directed against the core protein remained strongly positive (4+) in 87% of the patients (Fig. 2A–D). There was no correlation between changes in RIBA titres and patient characteristics (Table 3).

**Table 3:** Characteristics of the patients according to anti-hepatitis C virus ratio annual decrease

	≤11%	>11%	
Characteristic	*n*=77	*n*=80	*P*
Male, *n* (%)	46 (60%)	56 (70%)	0.858
Age (years)			
Mean ± SD	48 ± 11	45 ± 10	
Range	(31–79)	(20–78)	0.267
ALT level (IU/ml)			
Mean ± SD	129 ± 80	129 ± 88	
Range	(40–372)	(45–520)	0.648
Anti-HCV OD			
Mean ± SD	95 ± 21	92 ± 16	
Range	(28–126)	(39–123)	0.143
Mode of infection (*n*, %)			
Blood transfusion	23 (31)	29 (36)	
Injection drug use	26 (33)	24 (30)	0.750
Unknown	28 (36)	28 (34)	
Serum HCV-RNA			
Median (log_10_ IU/ml)	5.525	5.477	0.102
Range	(3.390–6.765)	(2.259–7.532)	
HCV genotype (*n*, %)			
1	30 (43)	34 (43)	
2	13 (18)	18 (23)	0.471
3	19 (28)	24 (30)	
4–5	8 (11)	3 (3)	
Fibrosis stage (*n*, %)*			
F0–F1	23 (36)	18 (28)	
F2	24 (37)	24 (38)	0.173
F3	10 (15)	16 (25)	
F4	8 (12)	6 (9)	
Pretreatment status (*n*, %)			
Naive	33 (43)	56 (57)	
Non-responders	17 (22)	15 (19)	0.433
Relapsers	27 (34)	19 (24)	

* Liver histology was graded according to the METAVIR scoring system: F0, no fibrosis; F1, portal fibrosis without septa; F2, portal fibrosis with rare septa; F3, numerous septa without cirrhosis; and F4, cirrhosis.

ALT, alanine aminotransferase; HCV, hepatitis C virus; OD, optical density; SD, standard deviation.

The median annual decrease was 11% from the initial ratio (mean ± SD 10 ± 12%; range 1–50%). In order to look for an association between the magnitude of anti-HCV ratio decrease and patients' baseline characteristics, the patients were classified into low (≤11%) decrease vs high (>11%) decrease (Table 3) with regard to the median annual anti-HCV ratio decrease. No association was found between patient characteristics and low vs high annual anti-HCV decrease.

**Fig. 3:**
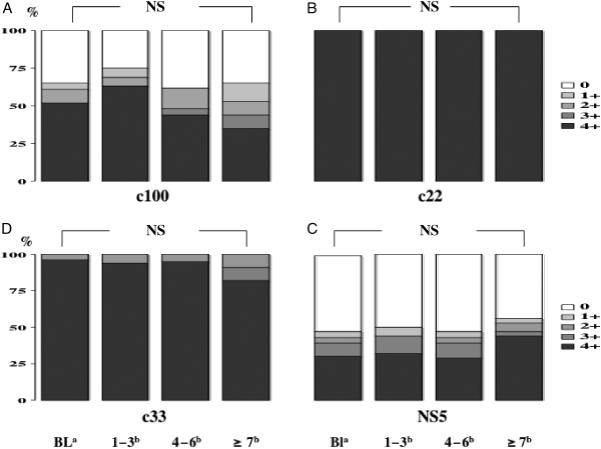
Changes in semi-quantitative hepatitis C virus (HCV) antibody titres [recombinant immunoblot assay (RIBA)] measured in 23 untreated patients with persistently normal alanine aminotransferase (ALT) and detectable HCV-RNA followed for a long-term period. ^a^Baseline. ^b^Years. (A) HCV antibody directed against NS4 protein (c100). (B) HCV antibody directed against core protein (c22). (C) HCV antibody directed against NS3 protein (c33). (D) HCV antibody directed against NS5 protein (NS5).

The characteristics of the 23 untreated patients with persistently normal ALT are shown in Table 1. The mean duration of follow-up was 6.18 ± 3.16 years (median 5.0; 4–13 years). Serum HCV-RNA remained detectable in all patients during follow-up. The mean OD was 65 ± 14 and 64 ± 19 in the first and the last measurements respectively (NS). RIBA titres and profiles remained unchanged during follow-up (Fig. 3A–D), contrary to patients with SVR.

**Fig. 4:**
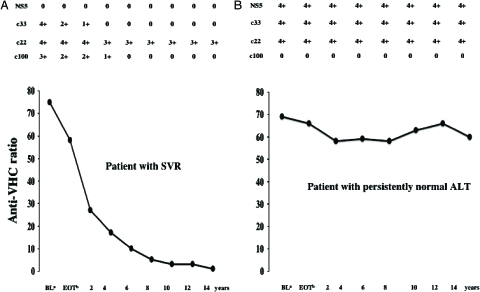
Long-term follow-up of the dynamics of alanine aminotransferase (HCV) antibody optical density (OD) and recombinant immunoblot assay (RIBA) profiles in one patient with sustained virological response (SVR) and one patient with persistently normal alanine aminotransferase (ALT) and detectable HCV-RNA. ^a^Baseline. ^b^End of treatment. (A) Patients with SVR (resolved infection). (B) Patient with persistently normal ALT and detectable serum HCV-RNA (ongoing infection).

Figure 4A and B shows the dynamics of OD and the RIBA-HCV profiles for one patient with an SVR and one untreated patient with persistently normal serum ALT.

## Discussion

Patients with an SVR are frequently lost to follow-up soon after therapy because these patients are considered cured and less apt to come for a post-treatment check-up. Therefore, regular and long-term follow-up of patients with SVR is not well documented, in particular, the dynamics of changes in the HCV antibody. This study confirms that there is no residual infection in serum in patients with SVR. On the basis of RIBA-HCV, there was a marked decrease in the antibodies directed to the non-structural proteins (NS3, NS4 and NS5), while the antibodies against the HCV core proteins (c22) remained strongly positive.

In the present study, none of the patients experienced a relapse during the follow-up period. The discrepancies between our results and other studies that report a relapse up to 5 years after treatment cessation may be because of the use of a less sensitive assay than TMA to assess the end-of-treatment response and thus overestimation of SVR (8–19, 20). Indeed, in our previous study (1), one out of 80 patients who achieved an SVR (assessed with an in-house polymerase chain reaction assay) developed a late relapse. An end-of-treatment serum sample from this patient was retested with TMA and demonstrated the presence of serum HCV-RNA (unpublished data), indicating that this patient was in fact a non-responder.

Although there are reports on changes in the HCV antibody or RIBA-HCV titres in patients who spontaneously resolve HCV infection (21), or soon after the end of therapy (22), there are very few reports on the long-term follow-up of the dynamics of changes in the HCV antibody after successful therapy (16–18). In our study, on the basis of RIBA, antibody against core protein remained strongly positive in most patients, whereas antibody against HCV non-structural proteins showed a marked decrease. The observation of longer persistence of core antibodies, in comparison with non-structural antibodies, could indicate that this structural protein involved in the constitution of the viral particle is synthesized in excess. Indeed, the structural core protein is the most immunogenic HCV polypeptide, and antibodies appear earlier than non-structural antibodies with a higher titre (23). A marked decrease was found in the antibodies directed against the NS3, NS4 and NS5 proteins, which have been reported to be associated with viral replication (NS3, NS5) (23–25), or to be highly immunogenic (NS4) (25), emphasizing the absence of ongoing antigenic stimulation in our patients with SVR. These results support the study by Nikolaeva *et al*. (23) showing that the humoral immune response to non-structural HCV proteins is short lived and might be a marker of infected cells. A decrease in the antibodies against the NS3, NS4 and NS5 proteins, observed in our study, could reflect a decrease in infected cells, suggesting that there is no antigenic stimulation after viral eradication. Furthermore, NS3 and NS5 antibody titres have been reported to be markers for chronicity (26) with a rapid significant decrease after treatment-induced viral eradication or in immunocompetent patients with self-resolved infection. This finding is supported by our previous study (27) showing significantly lower HCV antibody titres in untreated patients with persistently normal ALT and without detectable serum HCV-RNA (absence of ongoing infection) compared with those with detectable serum HCV-RNA (ongoing infection). Furthermore, our data are supported by the study by Takaki *et al*. (14) reporting a gradual decrease and disappearance in the HCV-specific antibody after recovery from HCV infection. Evidently, humoral immune response to non-structural proteins is short lived and might be a marker of infected cells destruction. Finally, the finding that HCV antibody titres of structural and non-structural proteins remained unchanged in untreated patients with persistently normal ALT and ongoing infection (detectable serum HCV-RNA) also supports our results.

In conclusion, this study is durable, associated with an absence of detectable serum HCV-RNA and a marked decrease in HCV antibodies, especially those directed against the non-structural NS3, NS4 and NS5 proteins. These results strongly suggest that HCV infection is cured in patients who achieve an SVR.

## Abbreviations

ALT: alanine aminotransferase
HCV: hepatitis C virus
PBMC: peripheral blood mononuclear cell
PEG-IFN: pegylated interferon
SVR: sustained virological response
TMA: transcription-mediated assay.
